# Are population level familial risks and germline genetics meeting each other?

**DOI:** 10.1186/s13053-023-00247-3

**Published:** 2023-03-08

**Authors:** Kari Hemminki, Xinjun Li, Asta Försti, Charis Eng

**Affiliations:** 1 Biomedical Center, Faculty of Medicine and Biomedical Center in Pilsen, Charles University in Prague, 30605 Pilsen, Czech Republic; 2 Division of Cancer Epidemiology, German Cancer Research Center (DKFZ), Im Neuenheimer Feld 580, 69120 Heidelberg, Germany; 3 Center for Primary Health Care Research, Lund University, 205 02 Malmö, Sweden; 4 Hopp Children’s Cancer Center (KiTZ), Heidelberg, Germany; 5 Division of Pediatric Neurooncology, German Cancer Research Center (DKFZ), German Cancer Consortium (DKTK), Heidelberg, Germany; 6 Genomic Medicine Institute, Lerner Research Institute, Cleveland Clinic; and Center for Personalized Genetic Healthcare, Cleveland Clinic Community Care and Population Health, Cleveland, USA

**Keywords:** Familial risk, Familial proportion, High-risk families, Nation-wide study, Family-Cancer Database

## Abstract

Large amounts of germline sequencing data have recently become available and we sought to compare these results with population-based family history data. Family studies are able to describe aggregation of any defined cancers in families. The Swedish Family-Cancer Database is the largest of its kind in the world, covering the Swedish families through nearly a century with all cancers in family members since the start of national cancer registration in 1958. The database allows estimation of familial risks, ages of cancer onset and the proportion of familial cancer in different family constellations. Here, we review the proportion of familial cancer for all common cancers and specify them based on the number of affected individuals. With the exception of a few cancers, age of onset of familial cancer is not different from all cancers combined. The highest proportions of familial cancer were found for prostate (26.4%), breast (17.5%) and colorectal (15.7%) cancers, but the proportions of high-risk families with multiple affected individuals were only 2.8%, 1% and 0.9%, respectively. A large sequencing study on female breast cancer found that *BRCA1* and *BRCA2* mutations could account for 2% of the cases (subtracting the proportions in healthy individuals) and that all germline mutations accounted for 5.6% of the cases. Early age of onset was a distinct feature of only *BRCA* mutations. In heritable colorectal cancer, Lynch syndrome genes dominate. Large studies on penetrance in Lynch syndrome have shown an approximately linear increase in risk from 40–50 years up to age 80 years. Interesting novel data revealed a strong modification of familial risk by unknown factors. High-risk germline genetics of prostate cancer is characterized by *BRCA* and other DNA repair genes. *HOXB13* encodes a transcription factor which contributes to germline risk of prostate cancer. A strong interaction was shown with a polymorphism in the *CIP2A* gene. The emerging germline landscape of common cancers can be reasonably accommodated by family data on these cancers as to high-risk proportions and age of onset.

## Introduction

Familial cancer has been the avenue for discovery of the first cancer predisposing genes which provided the scientific basis for clinical genetic counseling [1]. Although relatively rare, hereditary cancer became an essential part in advanced oncology clinics in response to the need to clinically action the high cancer risks conferred by germline mutations in predisposition genes [2–4]. The ultimate verification of heritable background requires mutation analysis, but high-risk individuals may show features that help their identification as carriers, including family history and patient-specific personal factors, such as age at diagnosis and tumor phenotype [2, 3]. While ascertainment of family history is still a part of the management recommendations, panel sequencing has been brought forward as a potential early diagnostic tool [5]. Multigene panels include a small or extensive battery of susceptibility genes, which allow detection of variants in multiple predisposing genes even in cancers which were previously considered gene-specific [6]. Use of panel sequencing has facilitated a comprehensive analysis of large patient cohorts covering wide age groups and, in some cases, including similar testing of healthy controls. The results have revealed the presence of pathogenic variants also in the control populations [7]. The extended sequencing in control populations show that, for example, pathogenic *BRCA v*ariants are not as rare as has been believed [8].

In the present article, we discuss recent results on germline genetics of common cancers and assess these in terms of the landscape of familial cancer described by the Swedish Family-Cancer Database. Eventually, understanding genetic background and cancer familial outcome have to converge, and the recent data from both appear complementary such that some uniform understanding may emerge. For this mini-review, the extensive literature on low-risk associations found in genome-wide association studies will not be covered.

### Familial cancer: how common and at what age?

We published recently a comprehensive study on the landscape of familial cancer, covering the population of Sweden over two generations (parental first generation and offspring second generation) [9]. The database includes Swedish families for close to a century and their cancers since 1958. Risks were calculated to the 20–84 year old second generation. Siblings could be identified only in the second generation. Screening and counselling for familial breast and colorectal cancers is in place in Sweden but the influence of these at the national level will take a long time, much past our study which covered cancers up to 2016 [9].

As cancer is largely an environmental disease, we have tried to estimate the environmental share in familial cancer risk by comparing familial risks with the risks between unrelated spouses and variation of familial risk in siblings by age difference and, for lung cancer, modelling based on heritability of the smoking habit [10–13]. These studies show that the sharing of smoking habit may explain some 20–30% of familial risk of lung cancer, and other sharing less for familial risks in gastric and testicular cancers; a small environmental component is likely for melanoma but a genetic background appears to be the main explanation for familial risks in other cancers.

Table 1 is a modification from the publication summarizing familial proportions for concordant cancers among first-degree relatives and the median diagnostic ages of the affected individuals in the second generation [9]. Only adult cancers were considered (diagnosis age over 19 years). For all cancer, the familial proportion was 13.2%, and it varied from the common cancers (26.4% of prostate cancer) to the rare cancers (0.2% in salivary gland cancers). Other cancers with high familial proportions were (female) breast (17.5%), colorectal (15.7%) and lung (13.0%) cancer. Statements about commonness of familial cancer are commonplace in the literature, almost invariably lacking referenced empirical evidence.

**Table 1:** Number of cancers, median ages, incidence rates, and number of familial cancer in the 20 to 84 year old index population (*N* = 9,338,882) during 1958–2016. Modified from ref. [9]

	Total numbers^a^			Family history of concordant cancer		
Cancer site	No	%	Median age	No	Familial proportion %	Median age
Lip	5772	1.1	60	119	2.1	61
Salivary gland	1299	0.2	53	3	0.2	49
Pharynx	3718	0.7	58	45	1.2	59
Esophagus	3854	0.7	63	73	1.9	63
Stomach	6980	1.3	61	411	5.9	62
Small intestine	2442	0.5	60	54	2.2	62
Colorectum	48,803	9.3	63	7650	15.7	63
Colon	30,819	5.9	64	3462	11.2	63
Rectum	17,984	3.4	62	1054	5.9	63
Liver, primary	4113	0.8	63	86	2.1	63
Pancreas	9557	1.8	64	465	4.9	63
Lung	33,121	6.3	64	4291	13.0	63
Breast	95,756	18.2	55	16,718	17.5	55
Cervix	10,542	2.0	40	223	2.1	39
Endometrium	16,012	3.0	61	821	5.1	60
Ovary	11,554	2.2	55	495	4.3	53
Prostate	91,696	17.5	66	24,238	26.4	65
Testis	8272	1.6	33	158	1.9	33
Kidney	8574	1.6	60	325	3.8	59
Bladder	18,738	3.6	64	1319	7.0	64
Melanoma	37,842	7.2	52	2715	7.2	52
Skin	19,014	3.6	67	1476	7.8	66
Eye	1607	0.3	57	10	0.6	58
Nervous system	21,560	4.1	51	790	3.7	52
Thyroid gland, adenocarcinoma	6044	1.2	42	136	2.3	41
Endocrine glands	11,713	2.2	53	341	2.9	50
Bone	1186	0.2	39	9	0.8	42
Connective tissue	3754	0.7	52	34	0.9	52
Hodgkins disease	4064	0.8	32	57	1.4	36
Non-Hodgkins lymphoma	17,197	3.3	59	743	4.3	60
Myeloma	5618	1.1	63	140	2.5	63
Leukemia	14,617	2.8	60	643	4.4	61
All above	525,019	100.0	60	69,104	13.2	62

^a^Total number of cancers was 552,953 including 27,934 diverse rare cancers, which were not included in this study.

The median age at diagnosis for any cancer in these second generation patients was 60 years (Table 1), which varied from early onset Hodgkin disease (32 years) and testicular cancer (33 years) to the late onset squamous cell skin cancer (67 years) and prostate cancer (66 years). The median age of onset of all familial cancer was 62 years, and only for a few cancer types, the familial cancer was of lower age of onset than for all cancer, namely, salivary gland (4 years less), endocrine gland tumors (3 years less) and ovarian cancer (2 years less). There is a technical reason which causes a small bias in this comparison. Familial patients are conditioned on family history, i.e., at least two members need to have the same cancer. As the follow-up time is limited, early onset patients would have a low chance of being familial. The bias should be even less in the analysis below, shown in the next table.

### High and low risk familial cancers

Familial standardized incidence ratios (SIRs ~ relative risk) for offspring of affected parents, depending on the number of affected family members, are shown in Table 2 [9]. The risks are calculated for offspring when one family member was diagnosed with the same cancer or when at least two members were affected (these families have at least three affected individuals, thus such families may be considered multiplex families). Some of the SIRs in multiplex families were very high and they were clearly elevated even for common familial cancers, i.e., 3.74 for prostate, 2.50 for breast and 2.76 for colorectal cancers (3.64 for colon cancer).

**Table 2:** Concordant familial risks when one or at least two probands were diagnosed with cancer. Modified from ref. [9]

	One family member diagnosed with concordant cancer					Two or more family members diagnosed with concordant cancer				
Cancer site	O	Mean age ± SD	SIR	95% CI		O	Mean age ± SD	SIR	95% CI	
Stomach	401	60.4 ± 11.1	**1.83**	**1.65**	**2.01**	10	62.0 ± 8.6	**5.55**	**2.64**	**10.25**
Colorectum	7233	61.5 ± 11.6	**1.70**	**1.66**	**1.74**	417	60.0 ± 12.0	**2.76**	**2.51**	**3.04**
Colon	3309	61.4 ± 11.6	**1.84**	**1.78**	**1.90**	153	59.3 ± 12.6	**3.64**	**3.08**	**4.26**
Rectum	1037	61.4 ± 10.4	**1.64**	**1.54**	**1.74**	17	59.2 ± 12.8	**2.20**	**1.28**	**3.52**
Pancreas	455	62.5 ± 10.0	**2.04**	**1.86**	**2.24**	10	53.1 ± 14.6	**4.96**	**2.36**	**9.15**
Lung	4122	62.6 ± 9.0	**2.11**	**2.05**	**2.17**	169	62.2 ± 8.8	**3.42**	**2.93**	**3.98**
Breast	15,805	55.0 ± 10.7	**1.74**	**1.71**	**1.76**	913	55.4 ± 10.7	**2.50**	**2.34**	**2.67**
Endometrium	799	59.5 ± 9.7	**1.90**	**1.77**	**2.03**	22	57.3 ± 9.0	**5.58**	**3.49**	**8.46**
Ovary	481	53.3 ± 10.8	**2.32**	**2.12**	**2.54**	14	51.6 ± 8.4	**8.99**	**4.90**	**15.13**
Prostate	21,688	64.4 ± 6.9	**2.20**	**2.17**	**2.23**	2550	63.5 ± 6.7	**3.74**	**3.60**	**3.89**
Kidney parenchyma	319	58.4 ± 10.8	**1.94**	**1.73**	**2.16**	6	34.5 ± 5.5	**5.17**	**1.86**	**11.33**
Bladder	1294	62.4 ± 10.5	**1.82**	**1.72**	**1.92**	25	63.0 ± 9.9	**2.48**	**1.60**	**3.67**
Melanoma	2620	51.3 ± 14.0	**2.41**	**2.32**	**2.50**	95	48.3 ± 14.4	**5.68**	**4.59**	**6.94**
Skin	1417	65.0 ± 10.2	**1.96**	**1.86**	**2.06**	59	65.7 ± 9.2	**4.60**	**3.50**	**5.94**
Nervous system	763	51.1 ± 14.6	**1.55**	**1.44**	**1.67**	27	39.3 ± 13.9	**6.24**	**4.11**	**9.09**
Endocrine glands	325	49.2 ± 14.0	**2.02**	**1.81**	**2.26**	16	46.9 ± 13.3	**14.82**	**8.45**	**24.11**
Non-Hodgkins lymphoma	737	58.5 ± 12.0	**1.71**	**1.59**	**1.84**	6	57.7 ± 12.6	1.51	0.54	3.31
Leukemia	626	59.3 ± 12.3	**1.88**	**1.73**	**2.03**	17	59.8 ± 7.3	**5.03**	**2.92**	**8.06**
All above	63,431	60.2 ± 10.8	**1.93**	**1.92**	**1.95**	4526	60.7 ± 9.9	**3.33**	**3.23**	**3.43**

*O* Observed, *SIR* Standardized incidence ratio, *CI* Confidence intervals

It is relevant to note that multiplex families covered only 6.7% of familial cancer. Combining data from the two tables, multiplex prostate cancer accounted for 2.8% of all prostate cancers; the multiplex share was 1% of all breast cancers, 0.9% for colorectal cancers and 0.5% for lung cancers.

We calculated the age of onset for cancer in the two types of families (bi-plex and multiplex) (Table 2). Overall, the mean age did not differ but for some cancers, the mean age of onset was clearly lower in multiplex families compared to 2-case families; the difference was about 10 years or more for pancreatic, kidney, and nervous system cancers, and it was 3 years lower for melanoma. For common cancers, the difference was 1.5 years for colorectal cancer, and 0.9 years for prostate cancer but there was no difference for breast and lung cancers.

High-risk genes for the germline genetic background for pancreatic and kidney cancers and for melanoma are well known and they are likely to contribute to the early diagnosis [6]. For nervous system cancers early onset gliomas and meningiomas are likely to contribute [14].

### Breast cancer genetics

A recent study on 60,000 patients with breast cancer and 53,000 controls using panel sequencing comprising 34 genes was able to shed novel details into the germline genetics of this cancer [15]. The mean diagnostic age was not given but it was probably somewhat lower than in an unselected population because many sub-cohorts had mean ages in the 40s or 50s. The sub-cohorts included population-based and family-based cohorts. Among truncating variants, 10 genes showed a significant (*p* < 0.05) odds ratio (OR) in the population-based studies, highest for *BRCA1* (10.57), *BRCA2* (5.85) and *PALB2* (5.02). Among missense variants, only three genes reached that significance level: the OR 1.42 for *CHEK2* and OR 1.1 for *BRCA1* and *RECQL*. In family-based studies, a slightly different set of truncating variants involving 11 genes was significant, including *PTEN* (OR 11.98) and *CDH1* (6.99); interestingly, risks for *BRCA1* and *BRCA2* were significant with ORs 2.77 and 2.75, respectively.

The modest ORs for many of the significant associations in this large study implied that the risk variants were found also in the healthy women and the frequencies were just barely below those in the cases. Large differences for protein truncating variants were observed for *BRCA2*, found in 1.5% of cases and 0.25% of controls, for *BRCA1*, 1.1% in cases and 0.1% in controls, and for *CHEK2*, 1.45% in cases and 0.6% in controls. The reported frequency of the *BRCA1* and *BRCA2* protein truncating variants in the control population translates to a variant frequency of 1:400 which has been found in an Australian study and in population databases [7, 8]. The authors concluded that 6.8% of the (European) breast cancer patients and 2.0% of the controls had protein truncating variants in the genes associated with breast cancer risk and 2.2% of the patients and 1.4% of the controls had missense variants in *CHEK2* [15]. The literature is full of overstatements about the contribution of *BRCA* to female breast cancer; the present figure of over 2% (removing the proportions in healthy individuals) should help to rectify understanding of breast cancer genetics (in the study population) with a small caveat about the age distribution of the study populations. Also the figure of 5.6% for known variants (again removing proportions for healthy individuals) is a justified reference figure.

Important findings revealed different age-related associations [15]. The ORs for BRCA1 and BRCA2 were highest at age < 40 years (32.8 and 11.9, respectively) and they declined systematically with age to 3.98 and 3.06 at age 60 + years. For 6 other genes (*ATM, BARD1, CHEK2, PALB2, RAD51C, RAD51D*), the age gradients were more even, and for *ATM* the highest risks were found at age 60 + years. With the exception of *BRCA*, for the 6 other genes more patients with the variants were diagnosed in the 60 + age group compared to < 40 age group. Although these results are not entirely novel, they reinforce the notion that for ‘high-risk genes’ age is an important dimension, also applying to any other genes.

### Colorectal cancer genetics

One of the first exome sequencing results were published on 626 UK familial colorectal cancers younger than 56 years in 2015 [16]. Lynch syndrome-related variants were found in 10.9% of the patients. Any pathogenic or likely pathogenic variants accounted for 14.2% of the patients. In this early-onset familial cohort, Lynch syndrome genes clearly dominated the germline background. However, even 32% of the patients without known deleterious variants had a first-degree relative with colorectal cancer, compared to 44% in gene carriers. Referring to Table 1, we can assume that early onset familial colorectal cancer accounts for less than 10% of all colorectal cancer. Thus, in that UK population, the proportion of Lynch syndrome of all colorectal cancer was most likely less than 1%.

Age-related cumulative incidence (CI, penetrance) for colorectal and other Lynch syndrome related cancers have been reported from ‘the Prospective Lynch Syndrome Database’ (PLSD) [17]. CI for pathogenic variants of *MLH1* was 25.0% by age 50 and it increased to 45.8% by age 75. For *MSH2*, the CI’s to age 50 and 75 were 19.4% and 43.0%, respectively, and for MSH6, they were 1.8% and 15.0%. For most other Lynch syndrome related cancers, the CIs increased relatively more after age 50 than in the case of colorectal cancers, i.e., their penetrance was shifted towards older ages. In a later study from the same database but with increasing number of patients, sex-specific CIs were reported with somewhat higher CIs for men than women among carriers of *MLH1* and *MSH2* mutations [18]. The CIs increased almost linearly from about 30 years to 75 years, except that for both *MSH6* and *PMS2* the increase in CI started at age about 50 years [18]. Relative risks for Lynch syndrome related colorectal cancer at 75 years were reported, and they were 12.1 for MLH1 carriers, 11.3 for MSH2 carriers and 3.9 for MSH6 carriers [17].

The International Mismatch Repair Consortium was able to gather data on 5255 families with Lynch syndrome and to use these to estimate risks (hazard ratios, HRs) and CI (penetrance) for *MLH1, MSH2, MSH6* and *PMS2* by sex, age and continental origin [19]. Almost all risks were higher in men than women. HRs varied extensively by the origin of the patients. Among Europeans, HR for *MLH1* was equal in young patients (diagnosis < 40 years) and older ones (> 60 years); in North American patients, the HRs were modestly (2–5 times) higher in young patients and in Australasian patients the HRs were 10 times higher in young patients. For MSH2, young patients had HRs doubled over old patients, but among non-Europeans they were up to tenfold in favor of the young patients. The CIs increased almost linearly from about 35–40 years to 80 years, except those for *MSH6* and *PMS2* where the increase in CI started at ~ 50 years [19]. The study found strong evidence for unknown risk factors in families that modified their risk depending on gene, sex and continent. For example, patients with specific *MLH1* and *MSH2* mutations were distributed among all deciles of CI between (0–10% and 90–100%). The possible modification of Lynch syndrome penetrance by the polygenic risk score has been tested with negative results [19].

### Prostate cancer genetics

Prostate cancer shows a high familial proportion, and its germline landscape is dominated by DNA repair genes in the two pathways, of homologous recombination (e.g., *BRCA1, BRCA2, ATM, BRIP1, CHEK2, NBN, BARD1, RAD51C, MRE11A,* and *PALB2*), and of mismatch repair (Lynch syndrome) [20]. An additional predisposing gene is *HOXB13* with a specific G84E mutation, which in the UK Biobank showed an OR of 4.81, in 1.29% of cases and 0.30% of controls [21]. The proportions of gene variants in 1662 US patients decreased in order *BRCA2* (3.8%), *ATM* (2.7%), *CHEK2* (2.5%), *HOXB13* (1.2%) *MSH2* (1.2%), *BRCA1* (0.8%), *MSH6* (0.8%) and 5 variants with smaller proportions all summing to 14.5% [20]. No data on healthy controls were reported and as variants are known to be present also in healthy controls the Figure 14.5% would be a large overestimation of the heritable background. The carriers of the variants were not distinguished from the non-carriers by age of onset, first-degree family history, family history of prostate cancer nor Gleason score.

The *HOXB13* gene encodes a homeobox transcription factor which is important in prostate organ development. The frequency of the mutation (polymorphism) is population dependent and it features early onset disease with high PSA levels. In Finland, the variant has a frequency of 1.8% and is associated with about 3.5-fold risk in prostate cancer; in Sweden, the variant frequency is lower but the risk is at the Finnish level [22]. The G84E variant showed a strong interaction with a *CIP2A* polymorphism in dual carriers; the OR for prostate cancer was 21.1 and the interaction was replicated in another Finnish cohort and with a lower risk in a Swedish population (OR 6.4) [15]. The *CIP2A* polymorphism alone did not influence prostate cancer risk. *CIP2A* is a cellular inhibitor of protein phosphatase 2A, a tumor suppressor in prostate cancer. One of the suggested mechanisms was HOXB13 protein binding to the *CIP2A* gene and promoting CIP2A transcription [15]. The dual carriers of these variants were very rare and the results, although significant, were based on small numbers.

### Familial risk and germline genetics

How did the results of our family study match the genetic results? We showed that early age of onset was not the feature of most familial cancers. Nevertheless, familial risks are high in early onset cancers, but for most cancers, the largest proportion of familial cases are diagnosed at over 70 years of age, with notable exceptions being breast cancer and melanoma [23, 24]. The new germline data on age-group specific sequencing appears to agree with the familial risk data, genotype relative risks were highest at young age when the non-genetic background incidence was low but at higher ages, the increasing background incidence attenuated or completely masked the genetic component. Although the guidelines for hereditary breast cancer and Lynch syndrome refer to age of 50 years as an important age limit, a large proportion of families are not caught by this age limit. The recent data on the penetrance of the Lynch syndrome genes show that the penetrance increases approximately linearly from an early threshold to 80 years, the highest age so far reported.

The question that logically follows is if the above observations weaken the predictive value of family history in genetic counselling and decisions for mutation analysis. Family history is most valuable in guiding to high-risk genetic background; confirming a family history in older patients (say over 70 years) may still be useful but the disease etiology is likely to be more complex than a verified germline mutation. The unknown familial component (discussed above), which contributes to risk in Lynch syndrome, may include genetic modifiers or familial environmental traits, such as dietary habits or gut microbiome, influencing the genetic traits [19, 25]. The other example was the interaction of the *HOXB13* G84E variant with a polymorphisms in the *CIP2A* gene, which panel sequencing would just read as a *HOXB13* variant [15].

Another main area of unmet knowledge is the magnitude of heritable cancers for which twin data are used as a kind of benchmark [26, 27]. Some recent ‘population-level’ studies are bringing substance to this area of previously unqualified statements of heritable etiology. In breast cancer, the figure of over 2% for the germline contribution of *BRCA1* and *BRCA2* is a justified estimate, as is 5.6% for all known variants (applicable to the populations used in the study) [15]. The Swedish family data showed that the proportion for familial breast cancer was 17.5%, and of these, 5.5% belonged to the multiplex families of at least three affected individuals (these were thus 1% of all breast cancer). One can assume that *BRCA*-related cancers constitute a large share of the cancers in the multiplex families but also contribute to the two-case group.

For Lynch syndrome, it is common to state that it accounts e.g., for 2.7% of colorectal cancer in Finland or 2.2–2.6% in Ohio, USA or 0.4% in Iceland, because these percentages of mutation carriers were found in large series of patients [28–30]. However no data were given for healthy controls and thus the likely etiological proportion of the pathogenic mutations will be less (cf. breast cancer study [15]). The Swedish multiplex families accounted for 0.9% of all for colorectal cancers. For prostate cancer, the penetrance estimates for the associated genes are incomplete but for the *HOXB13* variants the frequency is reported as > 4% in cases and 1.3% in controls [31]. In the Swedish family studies the multiplex prostate cancer families accounted for 2.8% of all prostate cancer.

## Conclusions

Large-scale sequencing of cancer patients has improved our understanding of the germline architecture of common cancers with increasing coherence with population-based family studies. The main novel aspects are qualified penetrance estimates, age-related risks and, not unexpectedly, documentation of deleterious variants for high-risk predisposition genes in apparently healthy populations. The belief that high-risk variants were very rare probably stemmed from sequencing of a few specific mutations in early onset patients or exaggerated familial cases only. The old wisdom is rectified also for germline genetics: never work without controls! We need to adjust the terminology of ‘gene X mutations contributing’ to the mutation frequencies in healthy populations.

## Data Availability

All data and material are available in the cited literature.
